# Idiosyncrasy

**Published:** 1885-04

**Authors:** 


### IDIOSYNCRASY.

When a peculiarity of constitution is manifested in a person, it is called an idiosyncrasy, provided it is of such a character as to be unnatural, or at variance with the *known* laws of nature.

Whether such a state of affairs really exists, viz., a condition contrary to the laws of nature, or whether it is merely a name given to something perfectly in accordance with nature, but which we cannot understand by reason of our lack of knowledge, is a disputed question.

Philosophers familiar with the inductive method of reasoning, hold that an idiosyncrasy arises from the action of perfectly natural causes, but believe that we are ignorant of the factors which enter into the problem. They believe that like causes produce similar effects, and deny that anything can happen which is at variance with the unchangeable laws of nature.

Others, however, think that in some instances like causes may, without reason, act differently in different cases, particularly in the living being, where there is such a complexity of organs, and such a number of elements entering into their construction, and where the proper performance of the functions depends on so many separate operations acting together to form a harmonious whole.

Whatever the explanation may be, the fact remains that idiosyncrasy does exist, and that it is not so rarely met with as some imagine. In certain subjects it takes the form of repugnance to certain articles of food; but this state is such a common one that it is scarcely classed under the term.

In the usual sense a person is said to have an idiosyncrasy who cannot take certain medicines, or upon whom they have a peculiar effect,

We know of two persons to whom the administration of an ordinary dose of morphine or chloral would bring certain death.

These cases are rare, but one can not tell when or where he will find them, so the importance of the precept, "Never give any poisonous medicine without the advice of a physician" is clearly seen.

Another form of idiosyncrasy is that in which there is a predisposition to disease, which develops in the course of time. As an example we have the disease known as the malignant pustule.

This comes from a peculiar state of the system, the poison being in the blood from birth. The pustule, which usually appears on the lips, or where the skin is tender, is the only indication that the system is in a diseased condition ; and when the pustule makes its appearance it is too late to effect a cure. Cancer in the same way indicates an idiosyncrasy of the system.

These last two diseases are, so far as our limited knowledge can determine, not hereditary, nor are they contracted ; but merely are the fulfillment and culmination of a so-called unnatural, and entirely unexplained phenomenon.

